# Purification and characterization of cysteine protease of *Sarcocystis fusiformis* from infected Egyptian water buffaloes

**DOI:** 10.1038/s41598-023-43147-1

**Published:** 2023-09-26

**Authors:** Amal Z. Barakat, Azza M. Abdel-Aty, Marwa K. Ibrahim, Hala A. Salah, Usama M. Hegazy, Rasha A. M. Azouz, Roqaya I. Bassuiny, Raafat M. Shaapan, Saleh A. Mohamed

**Affiliations:** 1 Molecular Biology Department, National Research Centre, Dokki, Cairo, Egypt; 2 Department of Microbial Biotechnology, National Research Centre, Dokki, Cairo, Egypt; 3 Zoonotic Disease Department, National Research Centre, Dokki, Cairo, Egypt

**Keywords:** Biochemistry, Biological techniques

## Abstract

*Sarcocystis* spp. infects water buffaloes (*Bubalus bubalis*) causing sarcocystosis. In the present study, *Sarcocystis fusiformis* was recognized in Egyptian water buffaloes based on histological observation and molecular analysis of internal transcribed spacer 1 (ITS1), *18S ribosomal* RNA (18S rRNA) and cytochrome c oxidase subunit I (COX-1) gene fragments. Chemotherapy and vaccines against *Sarcocystis* spp. could potentially target proteases because they may play a crucial role in the infection. Cysteine proteases are multifunctional enzymes involved in vital metabolic processes. However, the involvement of proteases in *S. fusiform* infection has not yet been characterized. Here, the purification and study on some biochemical properties of protease isolated from cysts of *S. fusiform* were carried out. Protease with a molecular weight of 100 kDa was purified. LC–MS/MS analyzed the protein sequence of purified protease and the data suggested that the enzyme might be related to the cysteine protease. The purified protease exhibited maximum activity at pH 6 and a temperature of 50 °C. The Michaelis–Menten constant (K_m_), the maximum velocity (V_max_), and the turnover number (K_cat_) were determined. The complete inhibition effect of cysteine inhibitors indicated that the purified enzyme is a cysteine protease. The results suggested that *S. fusiform* proteolytic enzyme may be necessary for parasite survival in water buffaloes by digesting host tissues. Therefore, cysteine protease could be a suitable target for vaccinations.

## Introduction

Muscular sarcocystosis is an Infectious disease caused by Apicomplexan protozoa of the genus *Sarcocystis* spp. The life cycle of this genus involves two hosts including an intermediate host (herbivorous prey: asexual stage and cyst-forming in muscle) and the definitive host (carnivorous predator: intestinal sexual phase and mature oocysts development)^1^. More than 200 species of Sarcocystis have been identified^2^, and they infect humans and a variety of domestic like cattle, buffaloes, sheep, pigs, and wildlife animals globally, leading to serious health and financial damages^3,4^. Sarcocystis-infected animals suffer from weight loss, low milk yield, anemia, miscarriage, and even death from severe infections^5^. Chronic sarcocystosis may lead to financial issues because of lessened meat, milk, and wool^6^ and the impact on human health due to the consumption of infected raw meat^5^. *Sarcocystis* spp. infection can result in mortality^7^. There are two types of Sarcocystis cysts, microscopic and macroscopic. The cysts are detected in the tongue, esophagus, diaphragm, heart, and skeletal muscles in the infected-Sarcocystis ruminants^8,9^. There are four species of Sarcocystis infecting water buffalo (*B. bubalis*) as intermediate hosts; *S. fusiformis* and *S*. *buffalonis*, *S.* *levinei,* and *S. dubeyi*. Cats are the definitive host for *S. fusiformis and S. buffalonis*, dogs are the definitive host for *S. levinei*, while *S. dubeyi* has not yet been identified^10^.

The overall prevalence rate of Sarcocystis infection in the examined slaughtered buffaloes at Egyptian abattoirs varies between 20 to 86% depending on hosts triated muscles, age, gender, geographic location/final host (cat and dog), size of cyst (microscopic and macroscopic), species and diagnostic method^9,11–14^. The high prevalence of Sarcocystosis in water buffaloes in Egypt is considered to be a genuine issue for both general health and the animal economy.

Parasitic enzymes which play an important role in host–parasite interactions and in the disease process, are attractive targets for diagnostic assay and vaccine development^15^. Proteases catalyze the hydrolysis of peptide bonds and they play important role in the development and maintenance of parasitic infections. Eukaryotic proteases can be classified into four catalytic classes: serine (e.g., trypsin, chymotrypsin, elastase), cysteine (thiol) (e.g., rennin, cathepsin) aspartic (e.g., pepsin) and metalloprotease (e.g., carboxypeptidase, thermolysin)^16,17^. Proteases have multiple functions for instance enable host invasion, tissue digestion and destruction, immune system evasion, clot dissociation and enhanced vascular permeability. Understanding the structural or functional relevance and substrate specificity of proteases can provide a basis for the discovery of new lead for antiparasitic therapy^18–21^.

The aim of the present work is to identify species of *Sarcocystis* spp. from Egyptian water buffaloes based on histological observation and molecular analysis. The purification and characterization of cysteine protease from identified *Sarcocystis fusiformis* is the second goal.

## Materials and methods

Experimental procedures were carried out in compliance with relevant guidelines. This study was approved by Medical Research Ethics Committee of National Research Centre, Cairo, Egypt (Subject N0. 1-3-1-5). The study is reported in accordance with ARRIVE guidelines (https://arriveguidelines.org).

### Specimen collection

Sarcocystis infected samples from the esophageal muscles of nineteen water buffalo (male, 3–5 years) were collected during slaughtering at Bassatin abattoir, Egypt, after being identified by the specialized veterinary doctor during the period from January 2021 to January 2022. Specimens containing macroscopic, sarcocysts were isolated directly. Morphological details were carried out by the naked eye and cysts were teased out by a fine forceps, washed with saline, and stored at − 20 °C for enzymes and DNA extractions.

### Histological examination

The infected muscles were formalin-buffered at 10%, dehydrated and paraffin-embedded. Hematoxylin (H) and eosin (E) were used to stain the muscle sections (5 μm). An Olympus CX 51 light-microscope was used to examine the stained muscles, and a C100-camera was used to take photomicrographs.

### Molecular analysis of sarcocystis

#### Genomic DNA extraction

Genomic DNA (gDNA) extraction was done using Thermo Scientific Gene JET kit, according to the manufacturer’s tissue protocol.

#### PCR amplification

The identification of *Sarcocystis* spp. was performed through PCR targeting the 18S ribosomal RNA (rRNA), mtDNA cyclo-oxygenase 1 (COX1) genes and Internal transcribed spacer1 (ITS1) rDNA gene region. We used pair primers of 18S ribosomal RNA (rRNA) (SF1/SR1) that are common for the genus Sarcocystis as previously described^22^ (Table 1). Primers for ITS-1 and COX1 genes were designed from the conserved regions in *S. fusiformis*, *S. buffalonis*, *S. levinei* and *S. sinensis* as detected by the alignment using GENtle software. The PCR reaction was conducted in a 50 µl-volume, containing 1 × Thermo Scientific Phusion HF buffer (1.5 mM MgCl_2_), 0.5 µM each primer, 50–150 ng DNA template, 1-unit Thermo Scientific Phusion DNA Polymerase and 200 µM dNTP in final reaction concentration. The thermal protocol of PCR was as follows: initial denaturation at 98 °C for 30 s, 30 cycles at 98 °C for-10 s, annealing at 60 °C for-30 s, and 72 °C for-30 s then a final extension step for-10 min at 72 °C. PCR amplicons were visualized by agarose gel electrophoresis (1.5%) with ethidium bromide. The DNA amplicons were purified from the agarose gels using Thermo Scientific GeneJET kit, Nanodrop spectrophotometric readings were used to quantify the results according to the manufacturer's recommendations. Sanger sequencing for the amplicons was performed by Macrogen (Seoul, South Korea) using the same primers as for PCR. The sequencing for 18SrRNA amplicons were done by new set specific for *S. fusiformis* (SF2/SR2) (Table 1).

**Table 1:** Primer sequences for PCR analysis.

Region of amplification	Forward	Reverse
18 S rRNA (SF1/SR1)	5′ GGCTGCATGTCTAAGTATAAG 3′	5′ GCCTCTAAGTGTTAAGGTTC 3′
18 S rRNA (SF2/SR2)	5′ TCTATGGCTAATACATGCGCA3′	5′ TCATCAAGTACACACCCTCACT3′
ITS1	5′ CAAGGTTTCCGTAGGTGAAC3′	5′ ACTGCGTCCTTCATCGTTGC 3′
*COX1*	5′ CTTACAACGTGCTGTTTAC3′	5′ GTCAACGGCCTCCGTATTCA 3′

### Enzyme activity assays

#### Caseinolytic-assay

The protease activity was assessed by employing the Lemos et al. method^23^ and azocasein as a nonspecific protease substrate. A reaction mixture (1 ml) containing 0.1% azocasein was mixed with a 20 mM Tris–HCl buffer solution with a pH of 7.2, and the mixture was then incubated at 37 °C for 1h. After incubation, 100 µl of TCA (20%) was added to stop the reaction, the mixture was then centrifuged at 12,000*g* for 5-min to remove the precipitate. An absorbance change of 0.1 at 366 nm is equivalent to one unit per hour.

#### Ninhydrin-assay

On the basis of the generation of free amino groups, the affinity of the isolated enzyme towards several native protease-substrates including casein, albumin, gelatin, collagen type I, hemoglobin, and fibrin was examined. The isolated enzyme (10U), 5 mg of each protease-substrate, and 50 mM Tris–HCl buffer, pH 6.0 were incubated at 50 °C. After one hour incubation, 100 µl of TCA (20%) was added to stop the reaction, the mixture was then centrifuged at 12,000*g* for 5-min to remove the precipitate. Following that, 100 µl of ninhydrin reagent and 0.5 ml of the reaction mixture were incubated at 84 °C for 5-min^24^. After cooling, absorbance at 570 was recorded. Isoleucine was employed as a standard, and the amount of amino acid liberated in µg/h was equivalent to one unit.

#### Cysteine-protease assay

The assay for cysteine protease was conducted using Arnon^25^ method. *N*-benzoyl-arginine-p-nitroanilide–HCl (BAPNA-HCl), a specific cysteine protease, was dispersed in 10 mM dimethyl-sulfoxide. One ml of the reaction mixture contains 100 µl of enzyme mixed with 800 µl of 50 mM sodium phosphate buffer, pH 6.0, containing 5 mM mercaptoethanol. The reaction started by adding 100 µl of 1 mM substrate followed by incubation of the mixture at 50 °C for 1 h. The cysteine protease activity was measured at 405 nm. One-Unit proteolytic activity defines as the amount of enzyme generating 1 µl mol of p-nitroaniline/h.

#### Determination of protein content

By employing the Bradford method, protein content was measured^26^.

### Purification of the protease from *S. fusiformis* cysts

#### Crude extract

The 400 mg of cysts were blended with a 50 mM Tris–HCl buffer (pH 7.0) and centrifuged in a cooled centrifuge for 15-min at 12,000 rpm. The supernatant was designed as crude extract and maintained at − 20 °C for subsequent examination.

#### Precipitation by ammonium-sulfate

In a cooling water bath, solid ammonium-sulfate was gently added to the crude extract until obtained 80% saturation. After centrifugation for 20-min at 12,000 rpm and 4 °C, the produced precipitate was dissolved and dialyzed against 50 mM Tris–HCl buffer, pH 7.0 at 4 °C overnight and centrifuged for 20-min at 12,000 rpm and 4 °C. The produced supernatant was designed as ammonium-sulphate fraction.

#### DEAE-Sepharose column

The DEAE-Sepharose column (15 × 1.6 cm) was equilibrated by 50 mM Tris–HCl buffer, pH 7.0 and then the ammonium-sulfate fraction was applied to it. At a flow rate of 60 ml/h, the enzyme was eluted utilizing a stepwise gradient from 0.0 to 0.5 M NaCl-in the same buffer, and fractions of 3 ml were compiled. The caseinolytic activity in the collected fractions was assessed, and the active peaks were pooled and stored at − 20 °C for further studies.

#### SDS-PAGE

The sodium dodecyl sulfate–polyacrylamide gel electrophoresis (SDS-PAGE) technique^27^ was utilized to assess the molecular mass and homogeneity of the obtained enzyme.

#### LC–MS/MS

The protein sequence analysis of protease from *S. fusiformis* was performed nano-Reverse Phase LC coupled to a QExactive Hybrid Quadrupole—Orbitrap mass spectrometer (Thermo Scientific, Bremen, Germany) through a nanoelectrospray ion source (ThermoScientific, Bremen, Germany) as following: a protein band of protease was in gel-digested for 3 h at 37 °C by trypsin. The resulting peptide mixture was resolved and applied on LC–MS/MS. Raw MS files were analyzed by the MaxQuant v1.5.3.3 proteomics software package. Precursor and MS/MS mass tolerance was set to 20 ppm for the first search (for the identification of the maximum number of peptides for mass and retention time calibration) and 4.5 ppm for the main search (for the refinement of the identifications). Protein and peptide false discovery rate (FDR) were set to 1%. FDR was calculated based on the number of spectra matched to peptides of a random proteome database (reversed sequence database) in relation to the number of spectra matching to the reference proteome. Peptide features were aligned between different runs and masses were matched (“match between runs” feature), with a match time window of 3 min and a mass alignment window of 20 min. Protein quantification was performed using the iBAQ algorithm through MaxQuant software.

### Biochemical assessment of the isolated-protease

#### Impact of pH and temperature on the isolated-protease

The characterization of the isolated enzyme was evaluated using the caseinolytic standard assay conditions as mentioned above^23^. The optimal pH was studied by applying 50 mM sodium acetate and Tris–HCl buffers with pH ranges of 5.0–6.0 and 7.0–9.0, respectively, for 1 h. The optimal temperature was studied by allowing the reaction mixture to be incubated at various temperatures ranging (25–70 °C) for 1 h. The impact of temperature on the stability of the enzyme was investigated. Before substrate addition, the enzyme was pre-incubated for 30-min at various temperatures ranging (25–70 °C), then cooled in an ice bath, and the residual activity was identified.

#### Kinetics parameters of the isolated-protease

The steady-state kinetic parameters of the isolated enzyme were studied at pH 6.0 and 50 °C with a range of azocasein concentration of 0.025 to 0.20 mg/ml, and incubation for 1h under the standard caseinolytic assay conditions. Michaelis–Menten plot using nonlinear regression analysis of the Graph-Pad prism program version 5 has been employed to determine the K_m_, and V_max_ values.

#### Impact of metal ions and protease inhibitors on the isolated-protease

Before adding substrate, the enzyme was individually pre-incubated for 30 min at 37 °C with metal ions at 2 and 5-mM final concentrations. For protease inhibitors, the reaction mixture included the BAPNA-HCl a specific cysteine substrate and inhibitor at 2 and 5-mM. The residual activity was then measured by the method of Arnon^25^. In the absence of an inhibitor or metal ion, proteolytic activity was considered to be 100%.

All experimental procedures were carried out in compliance with relevant guidelines.

## Results

### Macroscopic and histological examination

**Figure 1:**
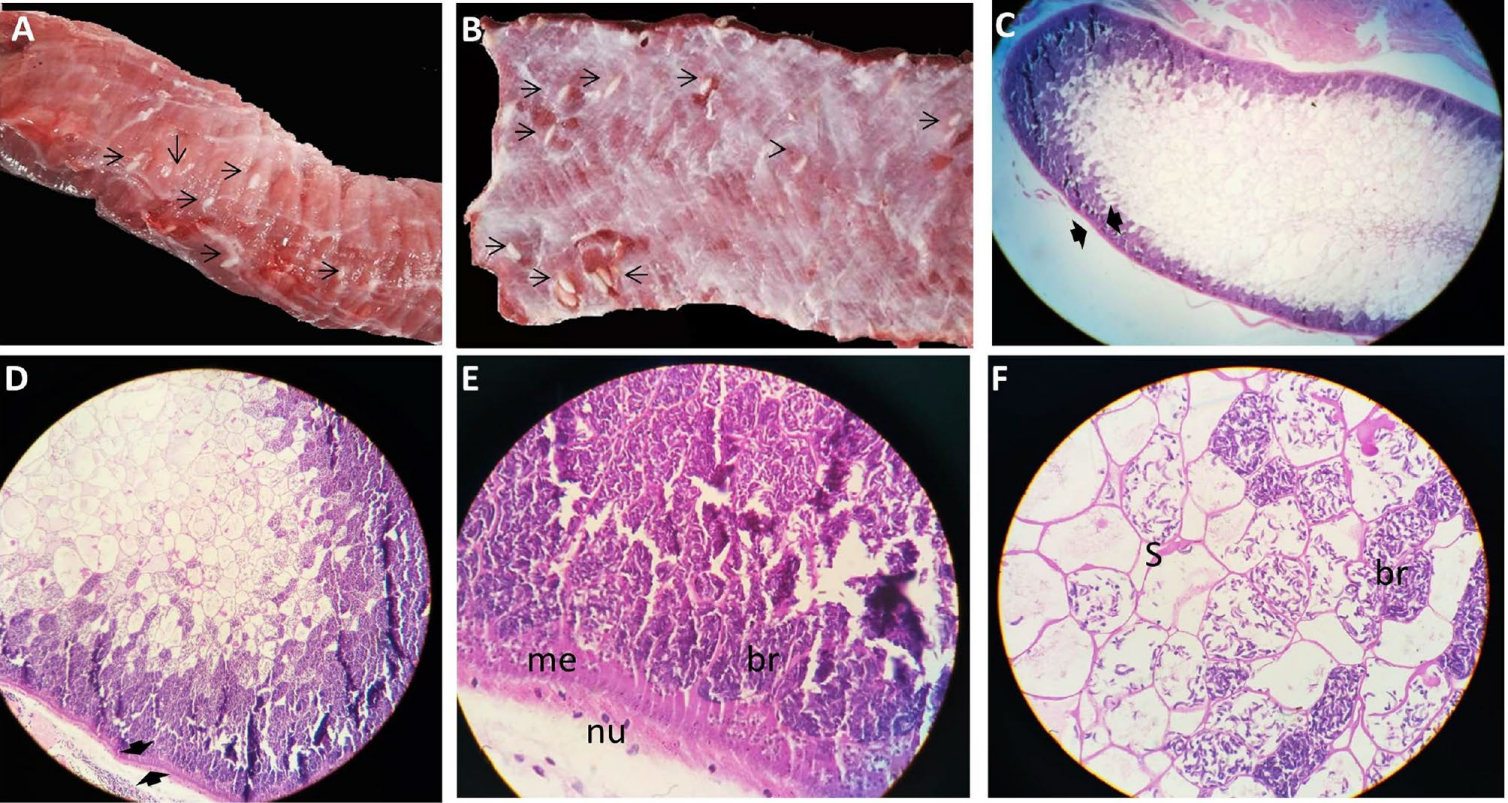
Macroscopical: oesophagus from water buffalo heavily infected with *Sarcocystis fusiformis*. The infection appeared as macroscopic spindle-shaped and milky white sarcocysts embedded in the esophagus muscles (**A**,**B**). Histological: Section of sarcocyst stained with hematoxylin and eosin (**C**, × 40; **D**,**E** × 100; **F** × 400). Thin sarcocyst wall (opposing arrowheads), septa (S) groups of bradyzoites (br), and faint staining metrocytes (me).

The sarcocystis-infected samples from the esophagus were collected separately from 19 water buffaloes slaughtered at the El-Basaten abattoir in Cairo. All infected tissues collected from water buffaloes exhibited macroscopic cysts (Fig. 1A,B). The macroscopic cysts were milky white and opaque in color, with a fusiform shape and ranged in size from 5–15 mm long × 1–4 mm wide (n = 22). Hematoxylin and eosin staining for this macroscopic cyst is shown in Fig. 1C–F. The examination of the histological section has shown that the cyst was bordered by a smooth thin cyst wall and the cyst cavity was divided into typical compartments of different sizes by thin septa. Two parasitic stages were within the cysts, metrocytes that acquired a pale stain near the margin of the cyst and the slender curved bradyzoites which filled most of the cyst, tightly packed in the interior area of the cyst and showed a dark stain.

### Molecular analysis

**Figure 2:**
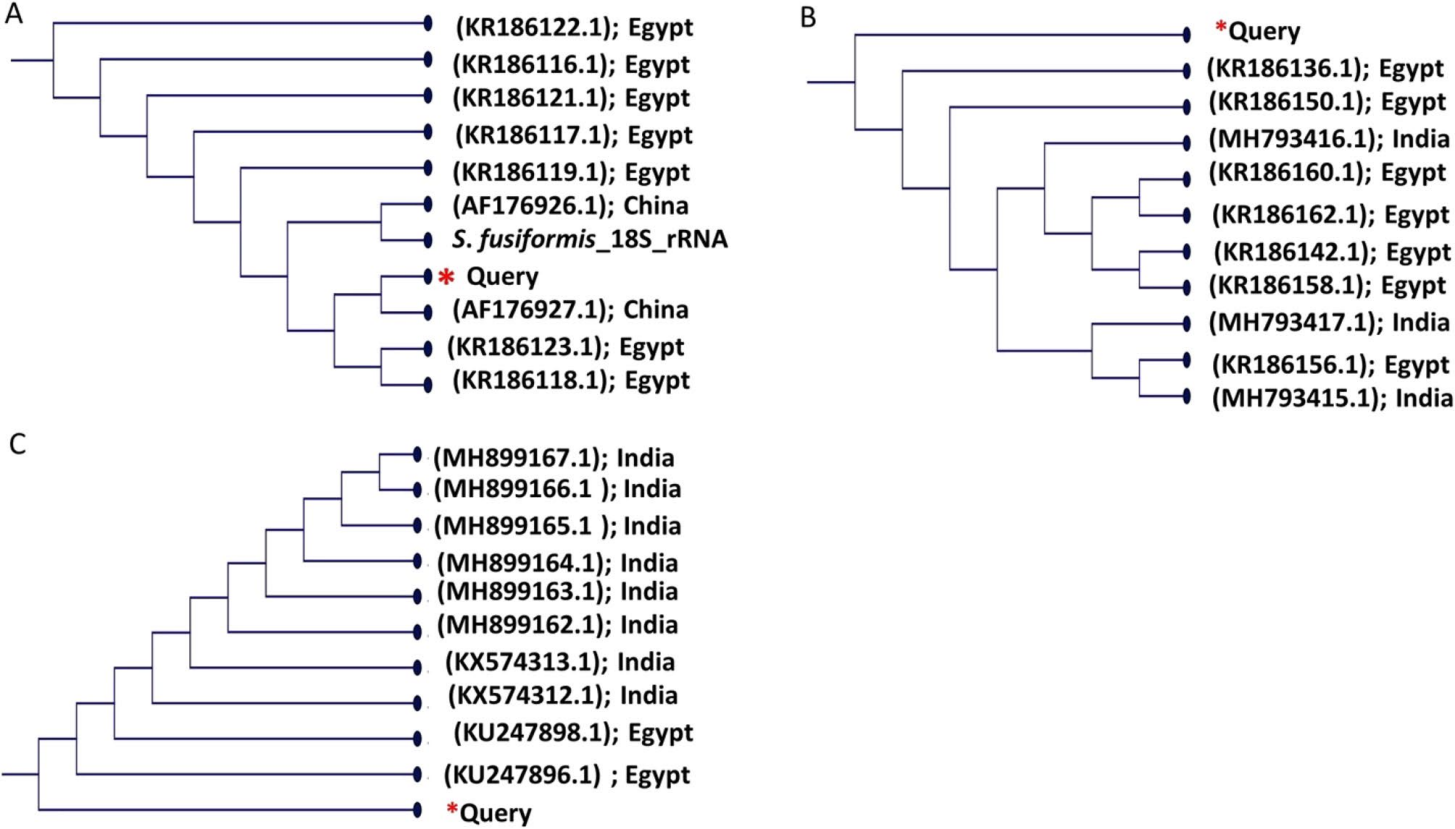
A phylogenetic tree for *Sarcocystis* spp. generated by a Fast Tree analysis using an NCBI alignment of (**A**) 18SrRNA, ITS1 (**B**), and COX1 (**C**) nucleotides. (*) Sarcocystis isolate.

**Figure 3:**
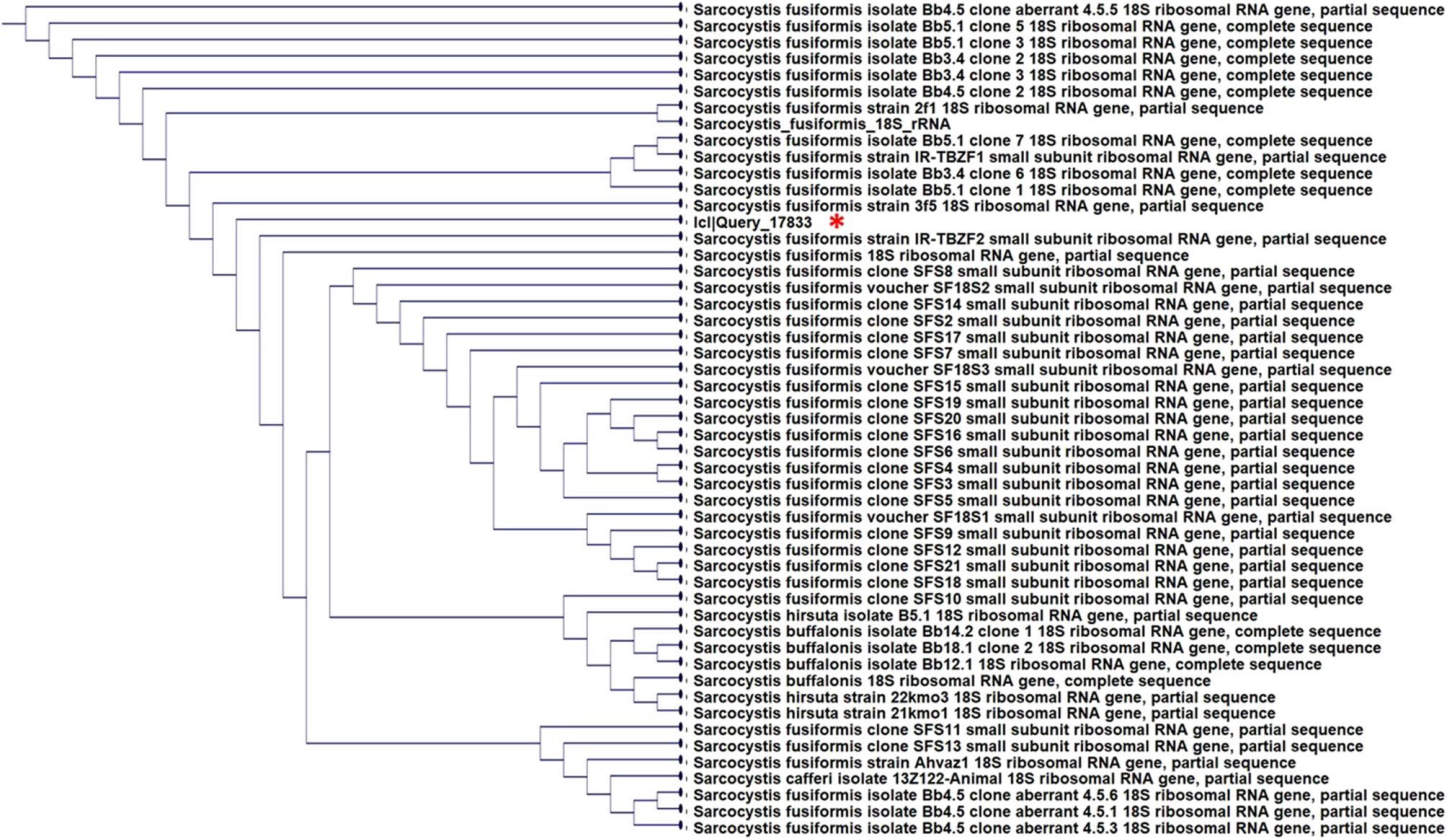
A phylogenetic tree form *Sarcocystis* spp. from water buffaloes generated by a FastTree analysis using an NCBI alignment of18SrRNA showing 50 clones.

The three selected genes (18S rRNA, ITS1, and mitochondrial COX1) were successfully amplified and sequenced from 15 isolates of sarcocystis. A BLAST comparative analysis of 18S rRNA gene amplicons with 1.8 kbp revealed that the isolates related to *Sarcocystis fusiformis* reference sequences with identity ranged from 89 to 97%. As the PCR products of 18S rRNA were amplified by a common primer for *Sarcocystis* spp., PCR amplification with specific primers for *S. fusiformis* was carried out, yielding PCR products of 553 bp for more conformation. The alignment of these partial 18S rDNA nucleotide sequences with that from others deposited in GenBank showed similarity ranging from 90 to 99% to *S. fusiformis*. The phylogenetic tree was generated based on the sequences with high identity (Fig. 2A). Our isolates are closely related to sequences from Egypt and China. The 700 bp-long ITS1 sequences of isolates showed 87–96% similarity to *S. fusiformis* in the GenBank, whereas the comparison of the 278 bp-long COX1 sequences of sarcocystis isolates exhibited 98–99% similarity to S*. fusiformis* sequences. Phylogenic trees based on ITS1 and COX1 were generated using some species with sequences having high percentages of identity (Fig. 2B,C). The later trees showed a high similarity to Indian and Egyptian isolates of *S. fusiformis*. A phylogenetic tree representing fifteen clones shows 48 clones of *S. fusiformis* and eight other closely related species (three *S. hirsuta*, four *S. buffalonis,* and one *S. cafferi*) (Fig. 3).

### Purification of the protease from *S. fusiformis* cyst

**Figure 4:**
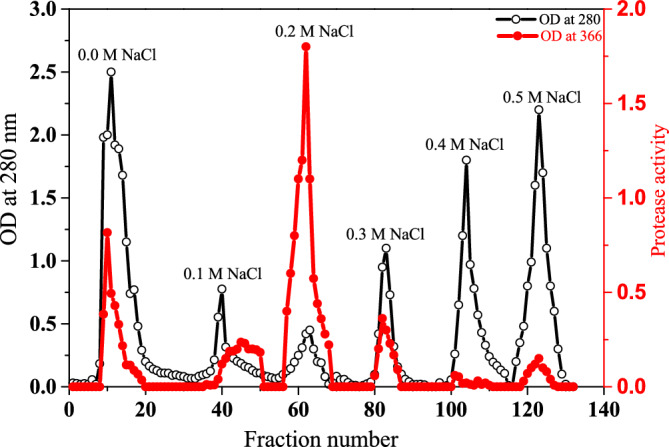
A typical chromatography profile of ammonium sulfate fraction of *Sarcocystis* spp. protease from infected water buffaloes on DEAE-Sepharose (15 × 1.6 cm) pre-equilibrated with 50 mM Tris–HCl buffer, pH 7.0. Three ml fractions were collected at a flow rate of 60 ml/h and 4 °C.

**Figure 5:**
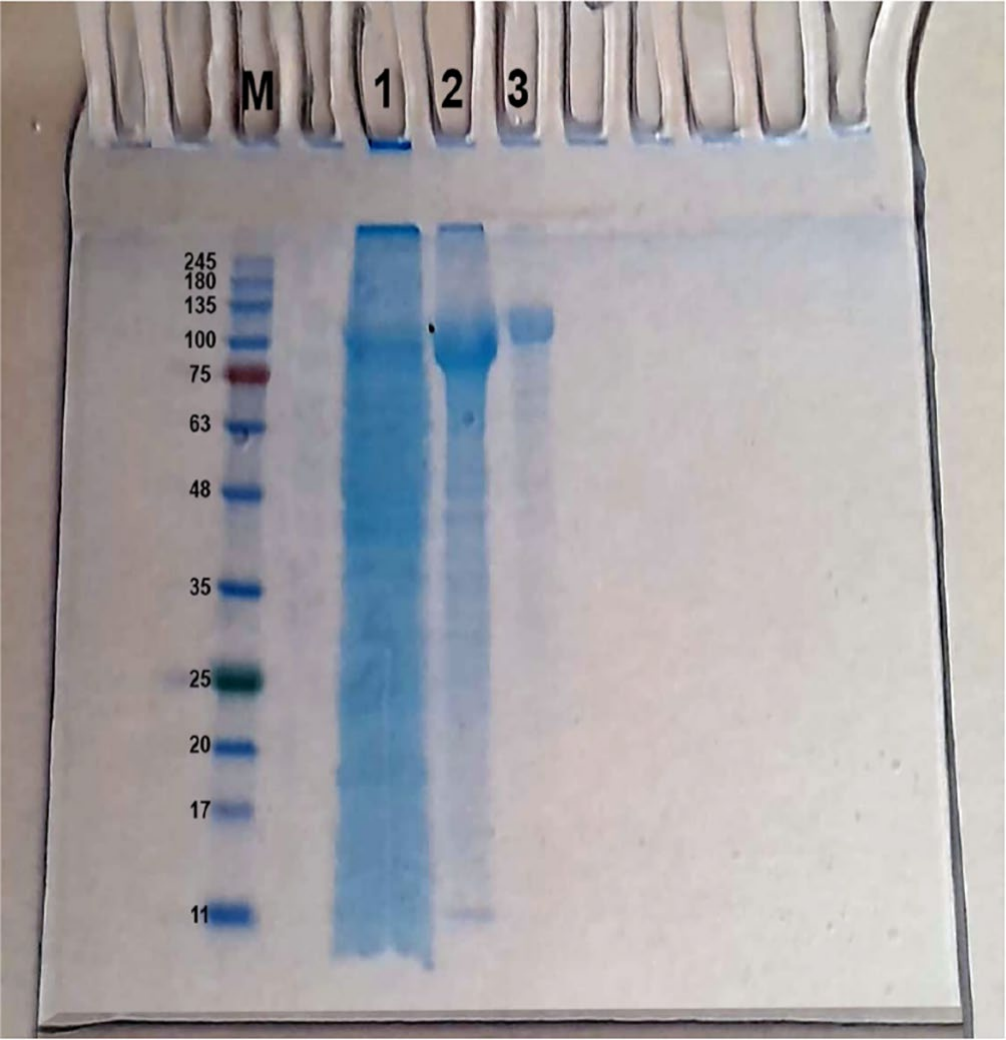
SDS-PAGE for homogeneity and molecular weight determination of protease from *Sarcocystis fusiformis*. (M) Protein markers; (1) Crude extract, (2) Ammonium sulfate fraction, (3) Purified enzyme of 0.2 M NaCl DEAE-Sepharose fraction.

The purification scheme of protease from *S. fusiformis* cysts is summarized in Table 2. Two steps were used to purify the enzyme. The initial step of purification is 80% ammonium sulfate precipitation. The dialyzed enzyme that was obtained from the ammonium sulfate process was loaded onto the DEAE-Sepharose column. When Tris–HCl buffer with varying concentrations of NaCl (0.0–0.5 M) was used as the elution buffer on DEAE-Sepharose, six peaks of enzyme activity were separated (Fig. 4). Separate peak-forming fractions were pooled, concentrated, and measured for protease activity. The 0.2 M NaCl eluted peak showed 20-fold purification and retained the major protease activity (as shown in Table 2). SDS-PAGE was used to verify the preparation's purity, and the peak representing protease protein migrated as a single band with a molecular weight of about 100 kDa, as shown in Fig. 5.

**Table 2:** Purification scheme of the proteases from the *Sarcocystis fusiformis*.

Purification step	Total protein (mg)	Total activity (U)	Specific activity (U/mg)	Purification fold	% Recovery	
					Protein	Activity
Crude extract	175	1770	10.1	1	100	100
Ammonium sulfate (80%)	104	1540	14.8	1.5	59.4	87
DEAE sepharose						
0.0 M NaCl	22	245	11.1	1.1	12.5	13.8
0.1 M NaCl	7.6	120	15.8	1.6	4.3	6.7
0.2 M NaCl	3.8	755	200	20	2.1	42.6
0.3 M NaCl	10	100	10.0	1.0	5.7	5.6
0.4 M NaCl	13	2.5	0.2	0.01	4.7	0.14
0.5 M NaCl	19	60	3.1	0.31	11	3.4

### LC–MS/MS analysis

SDS-PAGE protein band of protease from *S. fusiformis* was cut, tryptic digested and LC–MS/MS analyzed. The identified peptide fragments (Table 3), with posterior error probability score < 1% and protein/peptide false discovery rate < 1%, were aligned against *Toxoplasma* sp. proteases. Five peptide fragments out of 18 identified peptide fragments are highly similar to the N- and C-terminal ends of *Toxoplasma* sp. Cysteine protease and ovarian tumor unit domain-containing cysteine proteases. Out of these peptides 3 long peptides (ALVGVSASPKSGQVTSPFFVPLVGSPPYLSAYGEVETPAR, AVVASASSTSAFRLTGSAGVPFSDAASGGYVKTQDVSR and LGLAPGECWR) were homologous to a group of 16 OTU-like cysteine protease domain-containing proteins (group 1) of MW ~ 143 kDa and accession numbers of: A0A0F7USW2; S8GF61; S7UED7; A0A7J6K940; A0A425HSH9; A0A2G8Y620; A0A151HS29; A0A139XQK1; A0A125YX03; A0A086QNK3; A0A086PWS5; A0A086LU92; A0A086L465; A0A086JQ18; A0A086JLS6 and A0A2T6IPI8 (Fig. S1A). Similarly, one peptide (VDAAELDLDR) was homology to a second group of 22 OTU-like cysteine protease domain-containing proteins (group 2) of MW ~ 24 kDa and accession numbers of: A0A7J6KF34; A0A125YIL; S7VTI5; A0A125YIF9; A0A086PG20; V4YYX2; A0A086JH25; A0A086LJ08; S8EU58; A0A0F7UR23; A0A0F7UX68; S8F957; A0A7J6KDD8; A0A086JB35; A0A3R7YIL7; A0A2G8XM30; A0A139XIH1; A0A2T6IDA8; A0A151GYW9; A0A086PJ28; S7UHE2 and A0A086J6J6 (Fig. S1B). Finally, one peptide (VNAEALDLDR) was homology to a third group of 8 OTU-like cysteine protease domain-containing proteins (group 3) of MW ~ 22 kDa and accession numbers of: S7V2A4; A0A7J6K361; A0A3R8AIE5; A0A2T6J5G6; A0A086QEF7; A0A086LFR6; A0A086L2L9 and A0A086KVA5 (Fig. S1C). Collectively, these data suggested that protease from *S. fusiformis* cyst might be related to the OTU-like cysteine protease domain-containing proteins.

**Table 3:** LC–MS/MS analysis data for tryptic of the proteases from the *Sarcocystis fusiformis* peptides.

No.	M/Z of precursor ion	Charge^a^	Peptide sequence	Unique group^b^	PEP score^c^	Mass error^d^ (ppm)	Retention time^e^ (min)	MS/MS scan number^f^
1	1034.468	+ 1	ACAQGGSDLR	Yes	0.015	4.075	29.537	10,786
2	1354.712	+ 3	ALVGVSASPKSGQVTSPFFVPLVGSPPYLSAYGEVETPAR	Yes	0.012	–	47.671	20,240
3	618.3146	+ 6	AVVASASSTSAFRLTGSAGVPFSDAASGGYVKTQDVSR	Yes	0.015	− 4.0999	38.492	15,494
4	1488.432	+ 3	EESIPAVVLENTAYQLPTMPSLSSLTDGISPPKTWTPTVHR	Yes	0.012	–	45.208	15,909
5	1098.894	+ 3	LACVPRLMAATPGRSVSVQSGPGGEAEAVAAER	Yes	0.015	− 4.4326	39.756	15,537
6	606.1476	+ 7	LFPGENTTVFESPLSAQSILSCSPYNQGCDGGYPFLVGK	Yes	0.014	0.58933	52.391	22,467
7	1158.572	+ 1	LGLAPGECWR	Yes	0.014	3.9013	39.968	16,575
8	1207.908	+ 3	LLNYDENTHHPPATDPPSGSSFWSLGSPQASRR	No	0.013	− 1.0584	19.795	5672
9	967.2092	+ 4	PETASSFPSQASPAWALSSPPSVDSSSPAGASFSVASASR	Yes	0.013	− 3.1354	42.643	17,050
10	1134.271	+ 3	PTAQALSADSASAAARDRLLVFGVSFSPPLSAAR	Yes	0.010	–	39.234	15,273
11	754.3749	+ 5	QLQQDVTVVFSTWTHTLANWAAPWGAGPEEGGSVK	No	0.012	0.49563	33.929	12,585
12	1048.516	+ 4	QQSAERRGDCAGLDETPGSEQAVTDAPAYSYLILVLGPR	Yes	0.015	− 3.8063	42.025	17,351
13	488.2557	+ 2	QSLLQCAR	Yes	0.007	–	17.499	3823
14	620.9847	+ 6	RINEELLPQVHEVNVGAETVADFAEETVENLQK	Yes	0.010	− 4.4506	34.2	13,297
15	595.2814	+ 2	TAAENEENRR	No	0.011	3.3656	24.635	7533
16	725.6993	+ 6	TPMSQKSIYAASGPPSSLAPLERPWLSAPWCGLHSGELGGR	Yes	0.005	2.1497	37.687	14,350
17	558.7802	+ 2	VDAAELDLDR	Yes	0.009	− 0.52484	22.029	7151
18	558.2882	+ 2	VNAEALDLDR	Yes	0.011	− 0.13717	20.404	6223

^a^Charges are all charge states that have been observed. ^b^Unique group, when marked with ‘+’, is a unique particular peptide to a single protein group in the protein Groups file. ^c^PEP is Posterior Error Probability of the identification. This value essentially operates as a p-value, where smaller is more significant. ^d^Mass error (ppm) is mass error of the recalibrated mass-over-charge value of the precursor ion in comparison to the predicted monoisotopic mass of the identified peptide sequence in parts per million. ^e^Retention time (min) is the uncalibrated retention time in minutes in the elution profile of the precursor ion. ^f^MS/MS scan number is the RAW-file derived scan number of the MS/MS with the highest peptide identification score (the highest score is stored in the column ‘Score’).

### Biochemical characterization of isolated protease

#### Impact of pH and temperature on the stability and activity of isolated protease

**Figure 6:**
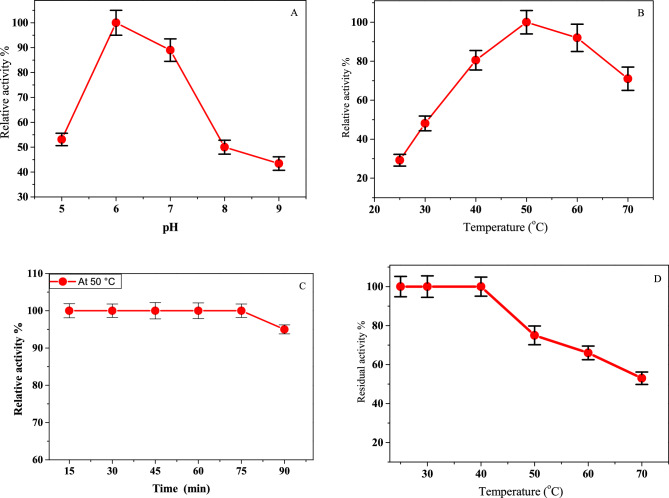
(**A**) Optimum-pH of the purified protease, (**B**) Optimum-temperature of the protease activity, (**C**) Relative activity % of the purified protease over different time periods (15–90 min) at 50 °C using the caseinolytic standard assay, (**D**) Thermal stability of the purified enzyme. The enzyme was incubated for 30 min at various temperatures (25–70 °C) before adding the substrate. After that, it was cooled in an ice bath. The residual activity was assessed using the caseinolytic standard assay. The values represent mean ± SD (n = 3).

Figure 6 depicts the influence of various pH and temperature values on the proteolytic activity of the purified 0.2 M NaCl-pooled fraction. The purified protease showed high enzyme activities in pH values ranging from 6.0 to 7.0 with an optimum pH of 6.0 (Fig. 6A). The purified protease displayed its optimal activity at 50 °C (Fig. 6B). And for confirmation, the enzyme activity has been measured over different time periods (15–90 min) at 50 °C using the caseinolytic standard assay. The results showed that the purified enzyme retained 100% of its activity when incubated with the substrate at 50 °C for 75 min then a very slight decrease (5%) was noticed at 90 min (Fig.6C). Therefore, this temperature (50 °C) was selected throughout the experiments as the standard temperature. For thermal stability, the purified enzyme maintained 100% activity when it was incubated alone for 30 min up to 40 °C; however, at 50 and 60 °C, a minor drop-in activity was observed (Fig. 6D). In addition, 47% of the enzyme's activity was still present at 70 °C. It could be concluded that the purified enzyme is a thermostable enzyme with optimal activity at 50 °C and pH 6.0.

#### Kinetics parameters of the purified enzyme

**Figure 7:**
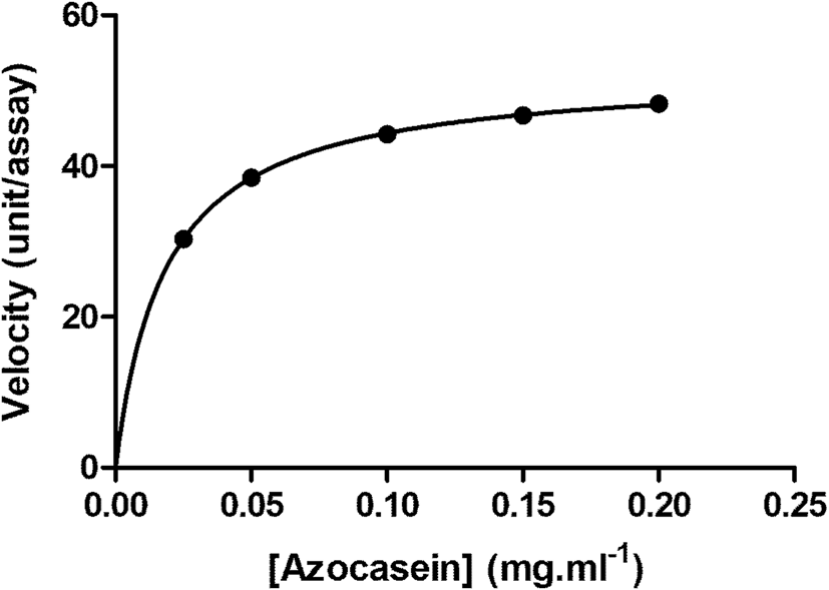
Michaelis–Menten plot of the purified protease using azocasein as a substrate at a concentration range of 0.025–0.20 mg/ml and reaction rate (velocity) has been measured under standard assay conditions.

Under ideal conditions (pH 6.0 and 50 °C), the kinetic properties of the purified-protease were examined using various substrate concentrations. Figure 7 shows the graphically determined Km, Vmax and K_cat_ values which were 0.018 mg/ml, 52.54 U/ml and 3.6 × 10^6^ S^−1^, respectively. A low K_m_ value indicates the high affinity of the purified-enzyme toward the substrate.

#### Impact of some metal ions and inhibitors on the enzyme activity

The activity of the purified enzyme was assessed in the presence of several protease inhibitors using a specific cysteine protease substrate (BAPNA-HCl) as shown in Table 4. At a concentration of 2 and 5 mM, the cysteine protease inhibitors, *p*-choromeruic benzoic acid, iodoacetic acid, and *N*-ethylmaleimide, totally suppressed the enzyme activity. While the metalloprotease-inhibitors (EDTA and *o*-phenanthroline) and serine protease-inhibitors (Soybean trypsin inhibitor and PMSF) have no inhibitory effect on the enzyme activity. From this finding, the purified-protease enzyme belongs to the cysteine protease group. Herein, the influence of various metal ions on enzyme activity was also investigated. At 2 and 5 mM, Zn^2+^, Ni^2+^, Hg^2+^, and Cu^2+^ act as potent inhibitors of the enzyme's activity, whereas the Ca^2+^ and Mg^2+^ have no impact on the enzyme's activity (Table 5).

**Table 4:** Effect of protease inhibitors on the proteolytic activity of the purified-protease using BAPNA-HCl, a specific cysteine substrate.

Inhibitor	Relative activity%	
	2 mM	5 mM
None	100 ± 1.1^a^	100 ± 1.9^a^
EDTA	99 ± 1.2^a^	97 ± 1.4^a^
*o*–Phenanthroline	98 ± 1.0^a^	97 ± 2.0^a^
Iodoacetic acid	2.1 ± 0.1^b^	0.0^b^
*p*-chloromercuribenzoic acid	0.0^b^	0.0^b^
*N*-Ethylmaleimide	1.8 ± 0.1^c^	0.0^b^
Soybean trypsin inhibitor	98 ± 0.8^a^	98 ± 0.2^a^
PMSF	98 ± 1.1^a^	97 ± 0.2^a^

Values represent mean ± SD (n = 3). Values with different superscript letters in the same column were significantly different at (*P* < 0.01).

**Table 5:** The impact of some metal ions on the proteolytic activity of the purified-protease from *Sarcocystis fusiformis*.

Metals	Relative activity%	
	2 mM	5 mM
None	100 ± 4.3^a^	100 ± 3.9^a^
Ca^2+^	100 ± 5.0^a^	100 ± 4.1^a^
Mg^2+^	99 ± 4.4^a^	98 ± 5.2^a^
Zn^2+^	0.00^b^	0.00^b^
Ni^2+^	14.4 ± 1.7^c^	10.8 ± 1.1^c^
Hg^2+^	0.0^b^	0.0^b^
Cu^2+^	0.0^b^	0.0^b^

Values represent mean ± SE (n = 3). Values with different superscript letters in the same column were significantly different at (*P* < 0.01).

#### Substrate specificity

The proteolytic activity of the purified enzyme was assessed in the presence of a variety of soluble and insoluble protein substrates (Table 6). A significant selectivity was observed for the insoluble substrates, including fibrin, native collagen I, and hemoglobin, with relative activity percentages of 100, 81, and 53%, respectively. For the soluble substrates, gelatin (denatured collagen) was preferable over casein and albumin with relative activity percentages of 43, 22, and 15%, respectively. This observation suggests that the purified protease has a high affinity towards blood and capillary wall components, resulting in disruption of the capillary wall and extravasations.

**Table 6:** Relative activity % of the purified protease from *Sarcocystis fusiformis* towards different native protein substrates.

Protein substrates	Relative activity (%)
Casein	22 ± 1.5^a^
Albumin	15 ± 1.0^b^
Gelatin	43 ± 2.2^c^
Fibrin	100 ± 5.4^d^
Collagen I	81 ± 5.6^e^
Hemoglobin	53 ± 2.5^f^

Values represent mean ± SD (n = 3). Values with different superscript letters were significantly different at (*P* < 0.01).

## Discussion

Numerous investigations revealed that the *Sarcocystis* spp. infection rate in sheep, cattle, and buffaloes is high worldwide^6,12–14,28–30^. Here, macroscopic and histological examinations were performed on 19 samples isolated from infected buffaloes collected at the El-Basateen slaughterhouse, Cairo province. The observation revealed that all animals were infected with *S. fusiforms*. Molecular analyses on the genetic characterization of diverse *Sarcocystis* spp. are usually carried out on target genes such as ITS, COX1, and 18S rRNA^31–33^. For accurate species identification, we used molecular techniques to confirm Sarcocystis species recognition in 15 samples. It is clear from the 18SrRNA, ITS1, and Cox1 phylogeny trees that the Sarcocystis found in Cairo province is identical to the sequences of *S. fusiformis*. Those sequences were previously reported in India, Egypt, and China. The characterization using the 18S gene revealed a marked similarity between the water buffalo species *S. fusiformis* and others in the Egyptian buffalo species^34^, the African buffalo species *S. cafferi,* and the cattle species *S. hirsute*^33^. *S. fusiformis* species was found in buffaloes in various Provinces throughout Egypt by several authors^9,13,34–36^. However, this species has been documented in water buffaloes over the world, including India^37,38^, China^39^, Iraqi^35^, Vietnam^40^ and Iran^41^. Current results and other previous findings suggest that *S. fusiformis* may be considered one of the most prevalent species of sarcocystis found in Egyptian buffaloes.

During the parasite's invasion of the host, proteases from protozoa perform a variety of functions. Dissolution of the extracellular matrix of the host is one of these, and it helps the parasite get to the surface of the host cell. In addition, protozoan proteases actively participate in the breakdown of host immune molecules such as immunoglobulins, making it possible for the pathogen to escape immune responses from the host. The protozoan protease also performs cytolysis and phagocytosis on target cells^42^. Cysteine proteases sometimes referred to as papain-like or thiol proteases possess a His/Cys catalytic dyad that interacts with one another. The cysteine (sulfhydryl group) initiates an attack on the peptide bond's carbonyl carbon during proteolysis by acting as a nucleophile. It has been discovered that parasitic protozoan cysteine proteases are involved in all of the major stages of disease development^43^. The merozoites of *S. muris* contained several basic and four acidic proteases. One of those basic proteases was further characterized as a thiol protease. The merozoite dense granules, the organelle involved in the parasite invasion of the host cell, were rich in thiol protease^44^. Therefore, the present study focused on purifying and characterizing proteases from *S. fusiforms* cysts. One-hundred kDa of protease was purified from *S. fusiform* cysts to homogeneity by two steps of purification with a yield of 42.6%.

This is the first study to our knowledge to describe the biochemical properties of the protease from *S. fusiformis*. Five identified peptide fragments of the purified protease-enzyme structure are highly similar to the N- and C-terminal ends of *Toxoplasma* cysteine protease and ovarian tumor unit domain-containing cysteine protease. This identification indicates that the purified protease belongs to the cysteine proteases. Additionally, the cysteine protease inhibitors completely blocked the purified enzyme activity, which was not impacted by serine and metalloprotease inhibitors. So, it is categorized as a cysteine protease. Similar results were reported for cysteine proteases from *Fasiola gigantica*^45^ and *Triticum aestivum*^46^. Further, the heavy metal ions Zn^2+^, Ni^2+^, Hg^2+^, and Cu^2+^ are effective enzyme inhibitors, whereas Ca^2+^ and Mg^2+^ have no impact on the enzyme's activity. Most of the tested heavy metal ions inhibited the cysteine proteases that were purified from *Fasiola gigantica*^45^ and *Triticum aestivum*^46^. Hg^2+^ poisoning is commonly attributed to the strong interactions that exist between Hg^2+^ and cysteine thiolate anions in cysteine proteases^47^. Moreover, the purified cysteine protease retained most activity in the pH range (6–7) and temperature range (40–50 °C). Several studies reported that *S. neurona* merozoites had serine protease activity with an optimal pH of 8 to 10 and a relative molecular weight of 65–70 kDa. And *S. neurona* merozoites were significantly prevented from entering cells by the serine inhibitors. *Toxoplasma gondii* had five genes encoding *cathepsins* (cysteine protease). Almost all *T*. *gondii* cathepsin proteases function best in low pH ranges (5.5–6.5), and they are differentially expressed in all parasitic stages of *T. gondii* (tachyzoites, bradyzoites, and sporozoites)^48–50^. *T*. *gondii* tachyzoite invasion was significantly reduced by PRT2253, a specific cysteine protease inhibitor of cathepsin-B^51^. Therefore, this similar characterization showed that *T. gondii* cysteine proteases were similar to *S. fusiform* cysteine proteases.

It has been reported that the optimum temperature of proteases from *Plasmodium knowlesi* and *Fasciola gigantica* was detected at 50 °C^52,53^. Additionally, it was shown that some Plasmodium parasite recombinant enzymes function best at temperatures between 50 and 60 °C^54^. The results of the current work observed that *S. fusiform* protease showed the same maximum catalytic activity at 50 °C. Interestingly, *S. fusiform* protease exhibited thermostability at a temperature of 40 °C and a slight decrease in activity at 50 °C for 30 min of incubation. It shares the character of thermostability with 80 kDa metalloprotease from *T. gondii*, possessing stability at 37 °C and a slight decrease at 56 °C for 30 min incubation^55^. Mathews et al.^56^ emphasized that the parasite encountered a series of thermal stresses during its entry into the host, like recurrent episodes of fever manifested in the host (reaching 41 °C or greater). The thermal stability of *S. fusiform* protease might be correlated with the parasite’s need to survive under extreme temperature changes in the environment. Further, the current study also indicated that the *S. fusiform* protease had a Km value of 0.018 mg azocasein/mL and Vmax value of 52.54 U/ml. In contrast, higher Km values were reported for proteases from *F. gigantica* (5 mg azocasein/ml)^52^ and microbe (3.8 azocasein/ml)^57^.

Current research demonstrated that *S. fusiform* protease effectively hydrolyzed several mammalian proteins, including fibrin and collagen I, albumin, hemoglobin, and gelatin. However, the enzyme showed strong activity against fibrin and collagen I. Sarcocystis forms observable cysts containing numerous undeveloped individuals. The cysts offer defense against the immune system, nourishment, and proper conditions of temperature, and hydration^7,58,59^. Several cathepsins from trematodes have anticoagulant potential due to their ability to effectively cleave fibrin and fibrinogen, promoting blood feeding^60^. They are also involved in the degradation of collagen, which is an important constituent of the host tissue^61^. Plasmodium parasites consume and digest host hemoglobin within the host cell niche to obtain the amino acids necessary for protein synthesis^62^. Falcipains, the major cysteine proteases of *P. falciparum*, were expressed in *P. falciparum* stages, and they efficiently hydrolyze hemoglobin at food vacuolar pH (approximately pH 5.5). *Falcipains* were impeded by cysteine protease inhibitors, which inhibit hemoglobin breakdown and parasite maturation^63^. Based on the displayed data, *S. fusiform* cysteine protease may be involved in invasion and intracellular growth.

## Conclusion

The current work reports the identification of *Sarcocystis fusiformis* from infected Egyptian water buffaloes based on histological observation and molecular analysis. The biochemical characterization of *S. fusiform* protease and the data on protein sequence suggested that protease might be related to the OTU-like cysteine protease. The results demonstrated unequivocally that the cysteine protease released by *S. fusiform* plays a crucial role in the behavior of parasites during tissue invasion, which is crucial for parasite survival. As a result, this enzyme may severely affect parasite viability if it is inactivated, making it a suitable target for chemotherapeutic drugs or vaccinations.

### Supplementary Information

Supplementary Figure S1.

## Data Availability

The datasets generated during and/or analyzed during the current study are available from the corresponding author on reasonable request.
