# Supplementary material for: Purification and characterization of cysteine protease of Sarcocystis fusiformis from infected Egyptian water buffaloes
Source: Sci Rep. 2023 Sep 26;13:16123. doi: 10.1038/s41598-023-43147-1 (PMC10522634; doi:10.1038/s41598-023-43147-1)
Supplement: Supplementary file 1 — Supplementary Figure S1. [file 41598_2023_43147_MOESM1_ESM.doc]

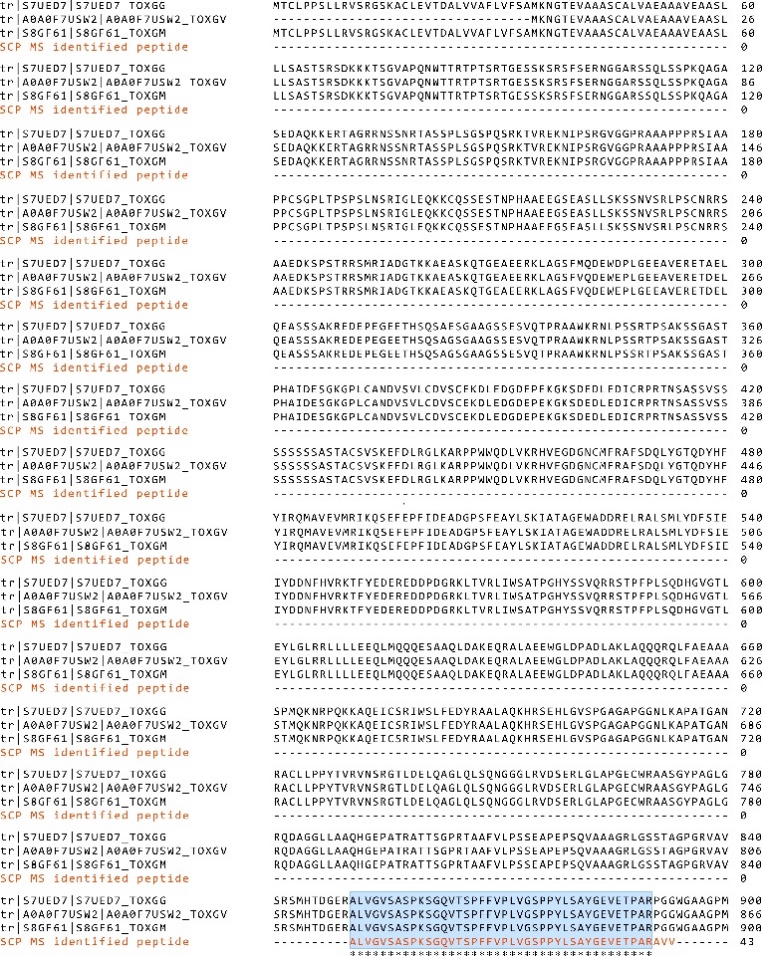


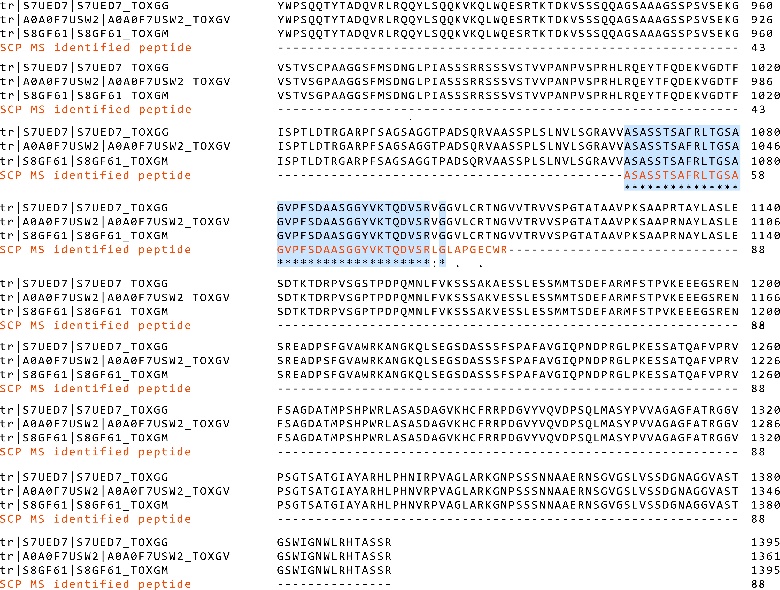


Figure S1A: Multiple sequence alignment of 3 peptides from tryptic digested protease from *Sarcocystis fusiformis* identified by LC-MS/MS analysis and a group of 16 OTU-like cysteine protease domain-containing proteins (group 1) of Mwt ~ 143 kDa. Three of these groups were selected with accession numbers: S7UED7, A0A0F7USW2 and S8GF61, respectively. The SCP peptides are shown in red and regions that share high homology are highlighted in blue. (*) indicates identical residues in all sequences, while (:) indicates the highly conserved residues and (.) for moderately conserved ones.


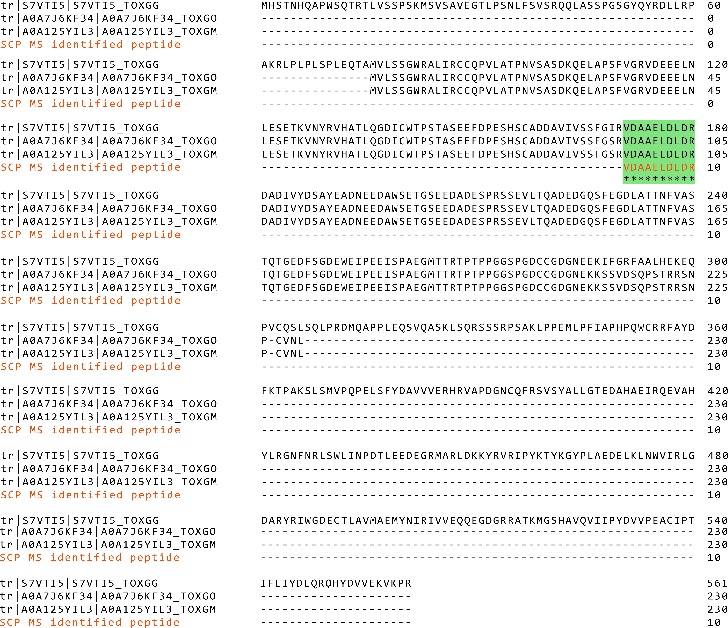


Figure S1B: Multiple sequence alignment of 1 peptides from tryptic digested protease from *Sarcocystis fusiformis* identified by LC-MS/MS analysis and a group of 22 OTU-like cysteine protease domain-containing proteins (group 2) of Mwt ~ 24 kDa. Three of these group were selected with accession numbers: S7VTI5, A0A7J6KF34 and A0A125YIL3, respectively. The SCP peptides are shown in red and regions that share high homology are highlighted in green. (*) indicates identical residues in all sequences, while (:) indicates the highly conserved residues and (.) for moderately conserved ones.


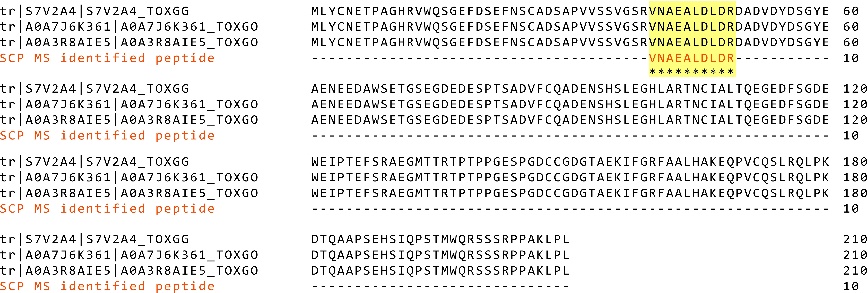


Figure S1C: Multiple sequence alignment of 1 peptides from tryptic digested protease from *Sarcocystis fusiformis* identified by LC-MS/MS analysis and a group of 22 OTU-like cysteine protease domain-containing proteins (group 3) of Mwt ~ 22 kDa. Three of these group were selected with accession numbers: S7V2A4, A0A7J6K361 and A0A3R8AIE5, respectively. The SCP peptides are shown in red and regions that share high homology are highlighted in yellow. (*) indicates identical residues in all sequences, while (:) indicates the highly conserved residues and (.) for moderately conserved ones.
